# The research progress of anti-inflammatory and anti-fibrosis treatment of chronic pancreatitis

**DOI:** 10.3389/fonc.2022.1050274

**Published:** 2022-11-24

**Authors:** Bing-Qing Li, Xin-Yuan Liu, Tao Mao, Tao-Hua Zheng, Peng Zhang, Qi Zhang, Yu Zhang, Xiao-Yu Li

**Affiliations:** Department of Gastroenterology, The Affiliated Hospital of Qingdao University, Qingdao, China

**Keywords:** chronic pancreatitis, anti-inflammatory agents, fibrosis, therapeutics, mesenchymal stem cell, phytochemicals

## Abstract

Chronic pancreatitis (CP) is a chronic progressive inflammatory disease of the pancreas, caused by multiple factors and accompanied by irreversible impairment of pancreatic internal and external secretory functions. Pathologically, atrophy of the pancreatic acini, tissue fibrosis or calcification, focal edema, inflammation, and necrosis are observed. Clinical manifestations include recurrent or persistent abdominal pain, diarrhea, emaciation, and diabetes. In addition, CP is prone to develop into pancreatic cancer(PC) due to persistent inflammation and fibrosis. The disease course is prolonged and the clinical prognosis is poor. Currently, clinical treatment of CP is still based on symptomatic treatment and there is a lack of effective etiological treatment. Encouragingly, experiments have shown that a variety of active substances have great potential in the etiological treatment of chronic pancreatitis. In this paper, we will review the pathogenesis of CP, as well as the research progress on anti-inflammatory and anti-fibrotic therapies, which will provide new ideas for the development of subsequent clinical studies and formulation of effective treatment programs, and help prevent CP from developing into pancreatic cancer and reduce the prevalence of PC as much as possible.

## 1 Introduction

Chronic pancreatitis (CP) is a pathological fibro-inflammatory disease of the pancreas that is characterized by persistent pathological reactions to internal and external injury or stress, resulting in intractable abdominal pain, pancreatic exocrine and endocrine dysfunction, decreased quality of life, and reduced life expectancy (1). The incidence and prevalence of CP are increasing, and its clinical prognosis is poor (2–4). In the world, the prevalence of CP is relatively stable, and over time, it has remained at about 35-100 per 100000 adults. The incidence rate increases year by year, about 5/100000 person years, and the incidence rate of males is twice as high as that of females (5). Among Asian countries, the statistical data in 2011 showed that the incidence rate in Japan was 14.0/100000 people, and the prevalence rate was 52.4/100000 people. The prevalence of CP in India is the highest in the world at 125/100000 people. In China, the prevalence rate of chronic pancreatitis was about 13.5/100000 people in 2003, and it was increasing year by year. At present, the latest incidence and prevalence of CP are under investigation (6). It is not clear whether the increase in epidemiological data really reflects a higher disease burden or simply a higher sensitivity of diagnostic tests (7).

CP is systematically classified according to the etiology of toxic, idiopathic, hereditary, autoimmune, recurrent, and obstructive pancreatitis (8). Some CP patients have acute pancreatitis, which is the initial event that starts the inflammatory process (9, 10). Subsequently, multiple risk factors such as pancreaticobiliary disease, alcohol, and smoking aggravate and maintain the inflammatory response of the pancreas. The pathogenesis of some idiopathic pancreatitis is often related to genetic variation, autoimmunity and other factors. In China, pancreaticobiliary obstruction is the main cause of CP (11, 12). In Western countries and Japan, 40–70% of CP cases are attributed to alcohol abuse (13). Alcohol metabolites directly damage acinar cells and play a role in promoting and maintaining chronic inflammation and fibrosis of the pancreas in CP (14). Smoking is an independent pathogenic factor of CP, with risk increasing in a dose-dependent manner (15).Another study have pointed out that the clinical characteristics of smoking related CP are different from CP with other etiologies. An independent chronic pancreatitis subtype should be added, which is caused by smoking related factors and has different characteristics from idiopathic pancreatitis or alcoholic pancreatitis (16).

The pathogenesis of CP is complex and involves changes in multiple protease- related genes. Loss of chymotrypsin C function and pancreatic secretory trypsin inhibitor (product of the SPINK1 gene) mutations increase the risk of CP (17, 18). PRSS1 (encoding cationic trypsinogen) mutation can reduce the risk of CP (19–21). The histopathological manifestations of the pancreas in patients with advanced CP are infiltration of inflammatory cells, deposition of extracellular matrix (ECM), atrophy of acinar structure, and replacement of fibrotic tissue, which gradually aggravate the clinical symptoms of CP and become an important reason for the poor prognosis of patients.

Pancreatic cancer (PC) is an important cause of cancer death, causing a serious health burden worldwide. CP has been proved to be a risk factor for PC. The persistent inflammation and fibrosis of CP are likely to develop into precancerous lesions of pancreatic cancer and eventually lead to pancreatic cancer. Therefore, it is very meaningful to intervene the disease at the early stage of CP to prevent its progression and deterioration.

At present, the main clinical treatment for CP is to relieve symptoms and prevent related complications. No specific treatment is available for the progression of CP (22, 23). However, *in vivo* and *in vitro* tests have shown that various active substances have achieved good results in targeting the etiology of CP. This paper summarizes the pathogenesis of inflammatory reactions and fibrosis during the course of CP, as well as the research progress on anti-inflammatory and anti- fibro tic treatments, in the hopes of promoting subsequent clinical research and formulate effective treatment plans.

## 2 Mechanism of CP inflammation and fibrosis and its development to PC

**Figure 1:**
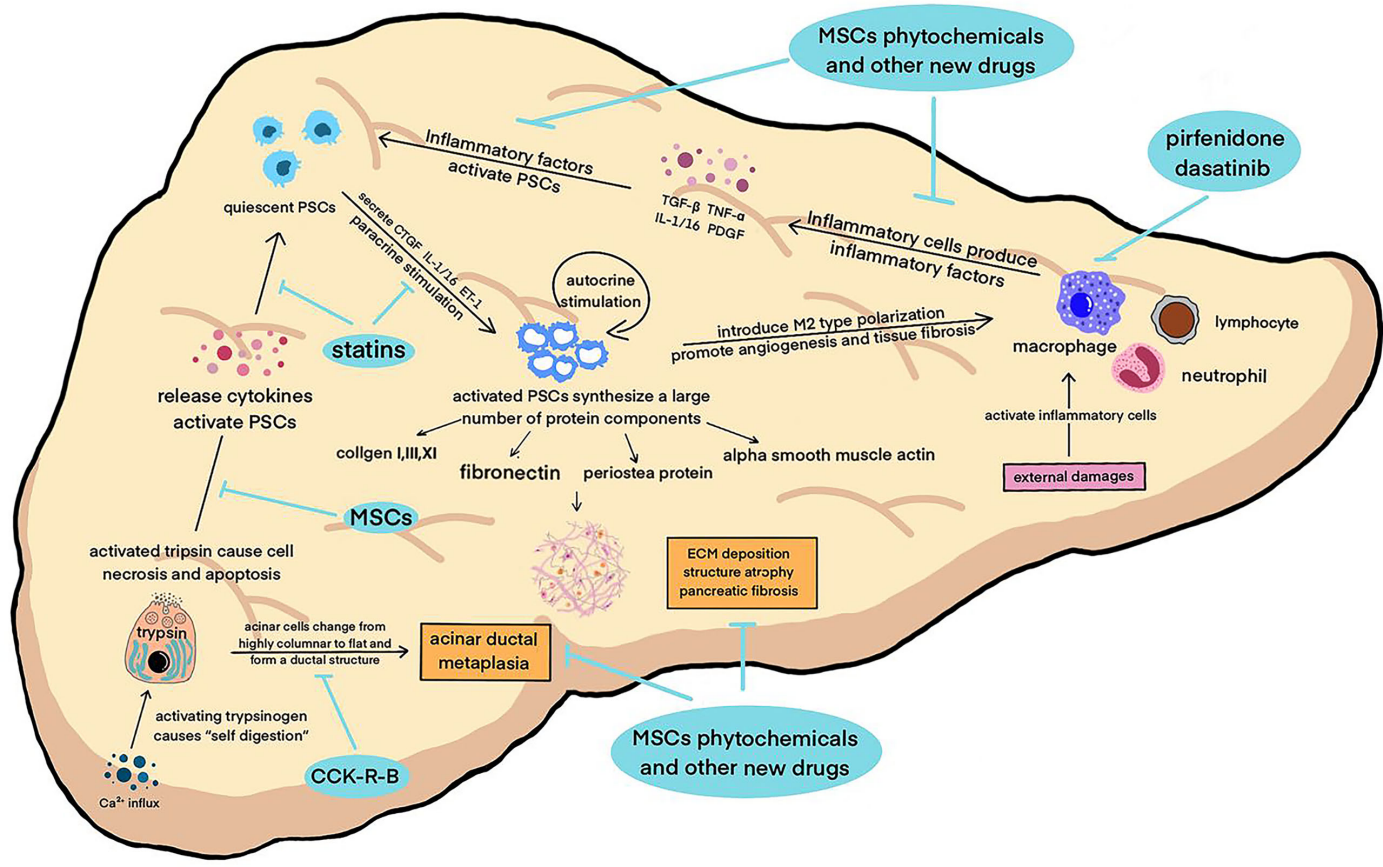
A variety of pathogenic factors damage the pancreas, activate macrophages and other inflammatory cells to secrete inflammatory factors, activate pancreatic enzymes in acinar cells at the same time, start “self digestion”, and lead to necrosis and apoptosis of tissue cells and the destruction of pancreatic structure. These factors work together to activate PSCs, which in turn can secrete inflammatory factors, aggravate the inflammatory reaction and cause a vicious circle. Activated PSCs can secrete smooth muscle protein, collagen fibers and other fibrosis mediators, leading to ECM deposition and pancreatic tissue fibrosis. The above pathophysiological processes continued to promote the progress of CP.

Inflammation and fibrosis of pancreas run through the whole course of CP. The activation of trypsin and other proteases, as the initial step of CP, damages acinar cells, further leading to the infiltration of multiple inflammatory cells such as macrophages (24). Immunocytes produce various types of pro-inflammatory cytokines that initiate the activation of pancreatic stellate cells. Recent studies have shown that the pathogenesis of CP involves changes in multiple protease- related genes, and inflammation and fibrosis of the pancreas are dynamic pathophysiological processes involving the regulation of multiple signaling pathways. Among them, pancreatic acinar cells, macrophages, and pancreatic stellate cells (PSCs) are key cells that jointly regulate the inflammatory and fibrotic processes of CP (25–27) (**Figure 1**).

### 2.1 PSCs play an important role in pancreatic fibrosis

Located in the periacinar region, with long cytoplasmic processes extending to the basolateral side of acinar cells, quiescent PSCs can maintain the integrity of pancreatic acinar cells and stabilize the synthesis and degradation of the ECM (28). Pancreatic injury can promote acinar cells, macrophages, and neutrophils to secrete inflammatory factors, such as transforming growth factor β (TGF-β), tumor necrosis factor α (TNF-α), Interleukin-1/16 (IL-1/16), and platelet-derived growth factor (PDGF), which rapidly activate PSCs (29). After being activated, PSCs can also secrete connective tissue growth factor (CTGF), IL-1/16, and endothelin-1 (ET-1), and then further promote the activation of PSCs through autocrine and paracrine signaling, which forms a vicious cycle (26). At present, TGF-β is considered the inflammatory factor with the strongest activation effect on PSCs. This effect is mainly achieved by regulating the extracellular signal- regulated protein kinase (ERK) and Smad2/3 signaling (30, 31). In addition, this mechanism also influences the phosphorylation level of three subtypes of the MAPK family, including c-Jun amino- terminal kinase (JNK), p38, and ERK (32–34).

Activated PSCs are characterized by the loss of perinuclear lipid droplets and active mitosis, and the endoplasmic reticulum is in a highly stressed state, showing high motility and contractility (35). The expression of multiple cytokine (e.g., PDGF and IL-1/16) receptors increases, and a large number of protein components constituting the ECM are synthesized, including collagen I, III, and XI fibronectin, and periosteal protein. PSCs develop a myofibroblast- like phenotype and express alpha smooth muscle actin (α-SMA), an activation marker. A large number of the ECM is deposited and degraded. These effects work together and gradually lead to pancreatic fibrosis (36–38).

### 2.2 Pancreatic acinar cells participate in the pathophysiological process of CP from multiple aspects

Researchers have proposed that intracellular calcium overload activates the NF- κβ signaling pathway, which then interacts with multiple signaling pathways to activate trypsinogen (39). Trypsin activation starts the “self- digestion” of acinar cells, and plasma membrane permeability is enhanced (24). Trypsin and other digestive enzymes cause necrosis and apoptosis of surrounding cells and destroy normal pancreatic tissue structure, resulting in pancreatic tissue atrophy (40). The dysfunction of acinar cells is another important factor in inducing the pathological response of CP, in addition to apoptosis and necrosis. A previous study confirmed that after the autophagic flow of acinar cells was destroyed, trypsinogen was activated and a large number of cytokines were released, which promoted PSC activation and initiated the process of fibrosis (41). At the same time, acinar cells in pancreatic tissue with CP gradually change from highly columnar to flat and form a ductal structure. This reaction is called acinar ductal metaplasia (ADM). Metaplastic acinar cells highly express cytokeratin (CK), and their molecular phenotype is very similar to that of pancreatic intraepithelial neoplasia (PanIN) cells. ADM is not only a promoter of pancreatic fibrosis but also a manifestation of it. It is considered an early event in pancreatic ductal adenocarcinoma (PDAC) prior to PanIN (42–45).

### 2.3 Inflammatory cells, such as macrophages, initiate and exacerbate inflammation and fibrosis in CP

The histopathological feature of CP is the infiltration of lymphocytes, neutrophils and macrophages, among which macrophages are considered to be the main inflammatory cells involved with fibrosis (46, 47). Previous studies have confirmed that necrosis and apoptosis of acinar cells can activate macrophages, which can induce PSC activation by releasing pro-inflammatory factors and create a positive feedback cycle by promoting PSCs to secrete more cytokines (48, 49). The latest research found that the polarization of macrophages affects the development direction of CP fibrosis. Activated PSCs can induce M2 (selectively activated macrophages) polarization of macrophages. M2- type macrophages have been shown to play a significant role in angiogenesis and promote tissue fibrosis (50–53).

### 2.4 CP develops to the precancerous lesion of PC and eventually to PC

During the development of CP to pancreatic cancer, we have observed the metastasis of pancreatic acinar cells, adm and PanIN. PanIN is defined as a non-invasive precancerous lesion. Its microstructure is papillary or flat, composed of columnar and cubic duct cells, and rich in mucus. They are classified into PanIN-1, PanIN-2 and PanIN-3 according to their degree of heterotypicity. PanIN usually exists in the pancreatic tissue near invasive pancreatic cancer and participates in the spread and metastasis of PC. Many case reports have confirmed that PanIN eventually develops into PC (54). The changes of cancer related genes have already appeared in pancreatic PanIN such as KRAS and TP53 mutations (55). Compared with other types of pancreatitis, hereditary CP is more prone to PanIN, which also suggests that the transformation of its pathological state is related to genetic factors.

When pancreatic injury persists, the inflammatory cascade is triggered, and a variety of inflammatory and pancreatic tissue cells interact to participate in the inflammatory and fibrotic responses of CP. Oxidative stress damage, autophagy, mitochondrial dysfunction, and endoplasmic reticulum stress have been shown to be multiple parallel mechanisms of CP pathogenesis (56). Similarly, in PC, the reaction of inflammatory cytokines leads to the proliferation of acinar, ductal and stellate cells, the transition from epithelial cells to mesenchymal cells, and progressive tumorigenesis (57). Understanding the pathogenesis will identify gene loci, specific cells, and downstream signaling pathways involved in CP and PC that can act as new treatment targets, providing favorable conditions for the discovery of specific drugs to prevent and delay fibrosis and disease deterioration (58).

## 3 Research progress of anti-inflammatory and anti-fibrotic therapies

### 3.1 Mesenchymal stem cells (MSCs)

MSCs are self-renewing pluripotent stem cells with multidirectional differentiation potential, and their application in organ transplantation and other aspects has been extensively explored (59, 60). MSCs have been shown to have remarkable therapeutic effects in a variety of diseases such as ulcerative colitis and idiopathic pulmonary fibrosis (61, 62)

The application of MSCs in CP can be summarized in two main aspects. First, MSCs can be used to restore pancreatic secretory function during total pancreatectomy and autologous islet transplantation (63). Second, MSCs have unique anti-inflammatory and immunomodulatory effects, which can reduce the inflammatory response during CP, inhibit fibrosis, and rescue pancreatic function (62, 64).

Here, we summarize the latest experimental results on MSCs for CP treatment (**Table 1**).

**Table 1:** Application of mesenchymal stem cells (MSCs) in animal models of chronic pancreatitis.

Source	Route	Animal	Method	Effect	Author
rat umbilical cord	vein	rat	DBTC	anti-inflammatory anti-fibrotic reduce pancreatic enzyme level homing of MSCs	Zhou.
fetal membrane	vein	rat	DBTC*	anti-inflammatory	Kawakubo.
adipose tissue	vein	mice	Ethanol Ceruloplasmin	anti-inflammatory anti-fibrotic increases pancreatic weight homing of MSCs	Sun.
adipose tissue	vein artery	rat	DBTC	anti-inflammatory reduces pancreatic cell apoptosis	Xu.
human umbilical cord	vein	rat	DBTC	anti-inflammatory anti-fibrotic reduces pancreatic cell apoptosis	Kong.
mouse bone marrow	vein	rat	L-arginine	anti-inflammatory anti-fibrotic improves pancreatic enzyme level reduces blood lipid level	Hager S.

*DBTC, Dibutyltin dichloride.

#### 3.1.1 Inhibits inflammation and pancreatic fibrosis

Studies have shown that a large number of inflammatory factors, such as IL-18, TNF-α, and growth factor TGF-B1 are significantly reduced in animal models of CP treated with MSCs, and tissue immune cell infiltration is alleviated. *In vitro* experiments also showed that when co-cultured with MSCs, the production of inflammatory factors in acinar cells and PSCs was inhibited and the inflammatory response of macrophages was reduced (65–70).

Pancreatic histopathology showed that MSC treatment improved collagen deposition, repaired damaged cells and Langerhans giant cells, reduced the expression of α-SMA, and reduced immune cell infiltration. Serum analysis showed that MSCs affected ECM metabolism by decreasing levels of hyaluronic acid and fibronectin (69, 70). Activation of PSCs is a key link that leads to pancreatic fibrosis. MSCs can downregulate the inflammatory response of PSCs and inhibit the activation of its downstream signaling molecules, indicating that MSC treatment can reduce pancreatic fibrosis by affecting PSC activity (67).

#### 3.1.2 Improves pancreas morphology and increases body weight

Compared to the CP group, mice injected with MSCs showed a significant increase in pancreatic volume and weight. Most animals with CP showed severe jaundice and decreased activity, while the general condition of MSC treated mice showed significant improvement. The body weight of the MSC group increased gradually and the total weight gain was greater than that of the control group (67).

#### 3.1.3 Improve pancreatic enzyme levels and reduce blood lipid levels

Compared with the untreated group, the decrease of protein expression and amylase activity in the pancreas during CP was reversed after MSC treatment, and abnormally elevated serum pancreatic enzyme levels decreased. Therefore, MSC treatment can improve exocrine function and attenuate the pancreatic injury caused by CP (70).

Compared with the CP group, serum total cholesterol (TC), triglycerides (TG), and low density lipoprotein cholesterol (LDL-C) levels significantly decreased and high density lipoprotein cholesterol (HDL-C) levels significantly increased in the MSC treatment group. Ahmed et al. proposed that normalization of the lipid profile of the MSC-treated subjects was achieved by improving β-c ell function and insulin resistance. MSCs can also secrete bioactive factors and differentiate into β-c ells to rescue pancreatic endocrine function (70, 71).

#### 3.1.4 Homing of MSCs and the differentiation into acinar cells

When inflammation occurs, MSCs respond to the signals sent by the tissues and are recruited to the inflamed or damaged parts requiring repair, which is called homing. A great number of labeled MSCs were found in the pancreatic tissue of the MSC group, whereas no MSCs were found in the pancreatic tissue of the control group (65). Moreover, most MSCs observed in the pancreas were amylase positive, indicating that MSCs not only migrated to the damaged pancreas but also differentiated into amylase acinar- like cells to repair the pancreatic tissue damage (67).

When stem cells are co-cultured with pancreatic acinar cells, they can differentiate into cells expressing amylopsin, and the differentiated cells had the same gene expression profile as pancreatic acinar cells, which indicates that MSC can repair damaged pancreatic tissue by directly differentiating into acinar cells (67).

#### 3.1.5 Reduces pancreatic cell apoptosis

Compared with healthy rats, apoptosis of pancreatic tissue cells in CP rats increased, which may be due to MSCs promoting the transcription and translation of apoptotic genes such as Bax, p53 and caspase-3. However, after MSC treatment, the expression of pro- apoptotic proteins decreased, and the expression of the anti-apoptotic protein Bcl-2 obviously increased, which indicates that MSCs could alleviate pancreatic injury by reducing pancreatic cell apoptosis (68, 69).

### 3.2 Phytochemicals

In recent years, phytochemicals have been widely used in clinical practice and have shown positive effects. Experiments have confirmed that phytochemicals have great potential in the treatment of CP and PC by reducing inflammation of the pancreas and slowing the progression of pancreatic fibrosis through a variety of different mechanisms and pathways (**Table 2**).

**Table 2:** The mechanisms and pathways of phytochemicals in the treatment of chronic pancreatitis.

Phytochemicals	Source	Mechanisms and pathways	Other diseases	Author
Curcumin	Curcuma	Relieves inflammation Inhibits the proliferation of pancreatic stellate cells (PSCs)	Cancer, Autoimmune, Lung diseases, CAD	Yang.
Rhein	Rhubarb	Inhibits the activation of PSCs Alleviates oxidative stress and endoplasmic reticulum stress	Tumors, Inflammation, Diabetic Nephropathy	O’Connell
Green tea catechin derivatives	Green tea	Inhibits the phenotypic transformation, proliferation, and migration of PSCs	Esophageal cancer, Stomach cancer, Periodontal diseases	Gorabi.
Resveratrol	Polygonum cuspidatum Vitis	Alleviates PSCs activation Reduces extracellular matrix deposition	Inflammatory bowel disease, Heart failure, Diabetes	Gundewar
Ellagic acid	Cruciferae	Inhibits the generation of reactive oxygen species Influences the expression of apoptotic genes	Allergic disease, Coronary disease	Liang.
Isoglycyrrhizin	Glycyrrhiza	Reduces macrophage infiltration Influences macrophage polarization Relieves inflammation	Endometriosis, Liver diseases, Cancers	Derosa.
Ganoderma lucidum polysaccharide	Ganoderma lucidum	Antioxidant Reduces the secretion of inflammatory factors	Tumor, ImmunoregulatoryDiabetic	Aslan.
Inonotus obliquus polysaccharide	Inonotus obliquus	Antioxidant	Cancers, Type 2 diabetes	Wang.
Apigenin	Celery	Induces apoptosis of PSCs Inhibits inflammatory signal transduction	Diabetes, Amnesia, Alzheimer’s disease	Wicks.
Olive leaf extract	Olive leaf	Reduces the level of inflammatory factors	Melanoma, Brain injury	Lu.

#### 3.2.1 Curcumin

Curcumin is an active substance with strong anti-inflammatory and anti-fibrosis effects, which is isolated and purified from Curcuma. In addition, polyphenols have antioxidant, antibacterial, anti- angiogenic, and anti-platelet aggregation properties. Because of these properties, curcumin can protect and prevent many diseases such as cancer, autoimmune diseases, lung, liver and cardiovascular diseases (72). It can improve liver fibrosis by inhibiting the proliferation of hepatic astrocytes, and improve myocardial fibrosis by modulating ECM metabolism (73).

*In vivo* experiments showed that curcumin inhibited the proliferation and activation of PSCs and decreased the expression of pro-fibrosis substances, such as type I collagen (Col I-α1) and fibronectin1 (FN1) in PSCs (74). As long as one tenth of the effective concentration of curcumin, curcumin analogues (L49H37) can induce PSC apoptosis, and its antiproliferative effect on PSCs is stronger than that of curcumin (75).

Curcumin has been tested on a variety of PC cell lines, and the results show that curcumin can induce apoptosis through EGFR downregulation and inhibition of NF-KB pathway. *In vivo* experiments show that curcumin and its analogues can inhibit angiogenesis and reduce the invasion of PC in mice, while liposome curcumin shows better effect. In addition, clinical trials have shown that oral curcumin is well tolerated in patients with PC and curcumin combined with gemcitabine shows better efficacy than alone (76).

However, the medicinal effects of curcumin may be overestimated, and no relevant drugs have been applied clinically to date. This may be related to the complexity of curcuma extract, which makes it difficult to purify the active components (77).

#### 3.2.2 Rhein

Rhein is extracted from the rhizome of rhubarb, which has many pharmacological properties, including anti-inflammatory and anti-angiogenesis effects (36). In a CP model, rhein alone or in combination with Salvia miltiorrhiza can significantly reduce the immune activity of α-SMA and TGF-β, the main activators of fibrosis, and inhibit the activation of PSCs, blocking a key node in fibrosis development. The deposition of ECM protein FN and Col I-α1 in the exocrine parenchyma also decreased. Researchers confirmed that this was mainly achieved by alleviating oxidative and endoplasmic reticulum stress and inhibiting SHH/GLI1 signal transduction (78).

#### 3.2.3 Green tea catechin derivatives

Epigallocatechin gallate (EGCG) is a phenolic compound extracted from green tea that exhibits strong antioxidant activity. EGCG inhibits the proliferation and migration of PSCs by inhibiting the PDGF- mediated signaling pathway. EGCG can also inhibit the transformation of ethanol- stimulated PSCs from a normal static phenotype to a myofibroblast- like phenotype (79, 80).

At present, research on EGCG is limited to the cellular level, and further animal experiments are needed to verify the role of EGCG in the treatment of CP.

#### 3.2.4 Resveratrol

When plants are attacked by external stimuli or pathogens, they will synthesize a polyphenol substance called resveratrol. Based on the current experimental results, resveratrol, similar to phenolic compounds such as rhein, can significantly impair the transcription and expression of several fibrosis mediators in PSCs and inhibit the proliferation and activation of PSC s. *In vitro* experiments showed that resveratrol could inhibit the proliferation, activation and migration of PSCs, which was initiated by reactive oxygen species (ROS). This may be related to the downregulation of microRNA 21 transcription levels (81).

Resveratrol improved the degree of pancreatic fibrosis in a CP model and effectively reduced PSC activation, ECM deposition, and the destruction of acinar structure in pancreatic tissue. The underlying mechanism may involve the accumulation of Mist1 in the nucleus and the inhibition of Akt and p38 MAPK signal transduction (81).

Previous studies have shown that resveratrol can inhibit the proliferation of PC cells, induce apoptosis and cell cycle arrest, inhibit the metastasis and invasion of cancer cells, and enhance the radiosensitivity of cancer cells (82). The latest experiment confirmed that resveratrol mainly affects NF- κ B signaling pathway to reduce the severity of pancreatitis and inhibit the occurrence of premalignant lesions such as ADM/PanINs, preventing the progression of PC (83).

#### 3.2.5 Ellagic acid

Ellagic acid is a natural polyphenol component widely existing in various soft fruits, nuts and other plant tissues which can reduce inflammation in chronic inflammatory diseases, such as Crohn’s disease and ulcerative colitis (84).

Studies have shown that ellagic acid can inhibit the activation of PSCs *in vitro* and prevent the differentiation of activated PSCs into fibroblasts (85). Rats treated with ellagic acid for ten weeks showed significantly reduced severity of pancreatitis and fibrosis. In addition, ellagic acid can inhibit the production of ROS in PSCs, which reduces TGF- β 1 and platelet growth factor levels (86).

#### 3.2.6 Isoglycyrrhizin

Isoglycyrrhizin is a bioactive component isolated from Glycyrrhiza roots and has been used for the prevention and treatment of cervical cancer, leukemia, and other diseases (87).

A previous experiment confirmed that after the treatment of CP rats with ILG, the pancreatic fibrosis was improved and the macrophage infiltration was significantly reduced. The follow-up team’s *in vitro* study on human PSCs showed that ILG could inhibit the activation and migration of human PSCs by downregulating the activities of ERK1/2 and JNK1/2. In addition, ILG weakens the M1 polarization trend of macrophages by affecting the NF-κB signaling pathway, but does not affect M2 polarization. The above experimental results suggest that ILG is a candidate drug for CP worthy of further study (88).

#### 3.2.7 Ganoderma lucidum polysaccharide

Ganoderma lucidum polysaccharide (GLP) is an important bioactive component extracted from *Ganoderma lucidum*, a common component of traditional Chinese medicine. It has been proved to have great health promoting value, such as anti-oxidative, anti-inflammatory, and anti-cancer effects (89, 90).

A study confirmed that three kinds of Ganoderma polysaccharides (GLPS3-I, -II, and -III) derived from fermentation broth, cultured mycelium, and fruiting bodies can significantly improve CP in mice. The mechanism of action mainly involves increasing the activity of antioxidant superoxide dismutase (SOD) and glutathione peroxidase, reducing the content of malondialdehyde (MDA), and reducing the levels of IL-1β and interferon-γ. In addition, GLPS3 - II extracted from mycelium performs better in the overall improvement of CP mice. It also changed the composition and diversity of intestinal flora and increases the proportion of beneficial bacteria. These results suggest that GLPS3 can affect CP development by changing the gut microflora (91, 92).

#### 3.2.8 Inonotus obliquus polysaccharide

Inonotus obliquus polysaccharide (IOP) is considered the main bioactive component of the medicinal mushroom Inonotus obliquus and has been shown to have anti-tumor and anti-oxidative properties (93).

Researchers purified it to obtain three polysaccharide components, IOP-I, II, and III, which were infused into a mouse model of CP. The results showed that purified IOP had pharmacological activity against CP, which mainly manifested as the slowing down of body weight loss, improvement of pancreatic fibrosis, and reduction in inflammation. When the dose of IOP was 400 mg/kg, optimal efficiency was achieved (94).

#### 3.2.9 Apigenin

Apigenin is a flavonoid that has many biological activities such as anti-tumor and cardio- cerebrovascular protection (95). A study showed that apigenin reduced the expression of collagen and fibronectin, reduced the pancreatic stress response to injury, retained acinar units, and alleviated acinar cell damage. With the prolongation of time and the increase of apigenin dosage, the viability of astrocytes gradually weakened and the apoptosis gradually increased. This may be inextricably related to the attenuation of the pro-inflammatory signaling pathway of TNF (96).

In addition, apigenin and its analogues showed significant efficacy in improving pancreatitis fibrosis even at very low doses (0.5 mg/kg) (97).

#### 3.2.10 Olive leaf extract

Olive leaf extracts improved CP in adult rats. The CP group showed a significant decrease in body weight and an increase in serum glucose, insulin, amylase, and lipase levels. A large increase in MDA levels and decrease in SOD levels were observed. After olive leaf extract treatment, the above parameters significantly improved, pancreatic degenerative changes were reduced, and TGF- β and IL-6 levels also decreased (98). There have been few reports of olive leaf extract improving CP, and further experiments are needed to confirm this.

### 3.3 Antioxidants

Antioxidants are commonly used in the clinical treatment of CP and are expected to alleviate persistent abdominal pain caused by inflammation and other pathological reactions (99). However, recent studies have shown that antioxidant treatment cannot reduce pain in patients with CP (100), nor can it improve the quality of life of patients in other respects (101). Whether the use of antioxidants can change the inflammatory state of CP or delay the pathological process remains controversial. Some studies have shown that antioxidant treatment can improve oxidative stress. However, other researchers have suggested that antioxidant supplementation can only increase the level of antioxidants in the body and has no practical significance in reducing inflammation, improving pancreatic fibrosis, and alleviating clinical symptoms (102). Interestingly, research on antioxidant therapy targeting the Nrf2 antioxidant pathway has yielded relatively consistent positive results. We will next focus on the research results on dimethyl fumarate, an Nrf2 activator, in the treatment of CP (103).

#### 3.3.1 Dimethyl fumarate

Dimethyl fumarate (DMF) alleviates ROS- induced cytotoxicity by activating Nrf2, which stimulates the production of glutathione (GSH), a reactive oxygen scavenger. DMF has been proven to have therapeutic effects on renal and pulmonary fibrosis (104). Recent studies on CP have suggested that DMF- treated rats had reduced pancreatic atrophy, acinar structure damage, and pancreatic histological severity scores; significantly improved glucose tolerance, and increased exocrine tissue volume. Continuous intake of DMF effectively improved histopathological abnormalities and islet cell function (105).

### 3.4 Other methods

#### 3.4.1 Pirfenidone

Pirfenidone is a pyridine ketone compound with a broad-spectrum anti-fibrotic effect that has shown good results in the treatment of idiopathic pulmonary fibrosis, liver fibrosis, and other fibrotic diseases (106, 107).

Pirfenidone ameliorates pancreatic tissue atrophy, acinar cell loss, and inflammatory responses when the pancreas is in a state of sustained injury. In *in vitro* experiments, pirfenidone reduced the profibrotic phenotype and infiltration of pancreatic macrophages and altered the cytokine environment in the pancreas before pathological changes occurred. No therapeutic effect of pirfenidone was observed in the absence of macrophages. In addition, pirfenidone has been shown to reduce collagen secretion, cytokine levels, and fibrosis marker expression in PSCs. These results indicate that this drug has great potential in the treatment of CP, but its efficacy as a therapeutic agent for CP needs further *in vivo* and *in vitro* tests and clinical trials (108).

#### 3.4.2 Dasatinib

Dasatinib is a multi- tyrosine kinase inhibitor commonly used in the treatment of acute and chronic leukemia (109). *In vitro* experiments confirmed that dasatinib had a significant inhibitory effect on the proliferation and activation of PSCs and M1 and M2 polarization of macrophages and hindered macrophage recruitment and crosstalk with PSCs. In a rat CP model, dasatinib also improved pancreatic fibrosis and reduced macrophage infiltration (110).

#### 3.4.3 Chemical pancreatectomy

Saleh et al. studied a method of “chemical resection of the pancreas,” which ablates pancreatic exocrine glands by flushing acetic acid through the pancreatic duct, prevents the inflammatory cascade caused by premature activation of pancreatic enzymes before other tissues of the pancreas are destroyed, and rescues the function of the damaged pancreas. They showed that after acetic acid treatment, inflammation of the pancreatic reserved tissue was reduced, the islet tissue was intact, pain was reduced, glucose tolerance improved, and insulin secretion increased. This operation can be easily performed in humans through endoscopic retrograde cholangiopancreatography. Dietary enzyme supplements are routinely used to replace exocrine function of the pancreas after surgery. However, chemical damage to the exocrine gland of the pancreas is an invasive procedure, and clinical experiments are needed to confirm safety (111).

#### 3.4.4 Cholecystokinin receptor antagonist

Cholecystokinin (CCK) is often used to induce experimental CP (112). Previous studies have shown that plasma CCK levels in CP patients are significantly elevated. It was also found that CCK could directly activate PSCs and induce collagen synthesis (113). After treatment with a CCK receptor blockers, the pancreatic weight of mice recovered faster and serum lipase levels decreased. Histopathology showed that inflammatory infiltration of the pancreas and metaplasia of the acinar ducts was reduced, which may be related to the decreased expression of genes related to pancreatic selective fibrosis (114).

#### 3.4.5 Replacement therapy improves the status of pancreatic FGF21 deficiency

FGF21 is a hormone secreted by the liver, and its main role is related to glucose metabolism, lipid metabolism and insulin resistance. In the CP model induced by chemical agents, the content of FGF21 was significantly increased (115). The onset of pancreatitis is initially induced by ATF4, with a concomitant increase in FGF21, which may indicate a preliminary attempt to overcome pancreatic tissue damage. However, with the persistence of pancreatitis, subsequent ATF3 induction effectively inhibits FGF21 transcription and plays an important role in contributing to CP. The expression of FGF21 is down regulated in a pancreatitis model. Supplementation with FGF21 can directly act on the exocrine function of the pancreas to reverse pancreatitis, which is manifested as a reduction in plasma amylase levels, alleviation of pancreatitis edema, and inflammatory infiltration and necrosis. The same effect can be achieved by blocking the induction of ATF3 and increasing the expression of FGF21 (116–118).

#### 3.4.6 Statins

Previous *in vitro* experiments have shown that lovastatin, a hydroxymethylglutaryl coenzyme A reductase inhibitor, can interfere with the activation of PSCs by blocking Ras signal. Because of its antioxidant characteristic, statins can reduce the production of interleukin-10(IL-10), alleviate pancreatitis and fibrosis, and improve exocrine function when applied to the rat model of CP. The combination of lisinopril and lovastatin can inhibit pancreatic fibrosis in rats with alcoholic CP after distal pancreatectomy, which is reflected in the down-regulation of PSCs activity (119). In addition, researchers confirmed that long-term use of statins can also prevent pancreatitis after endoscopic retrograde cholangiopancreatography(ERCP) (120).

At present, randomized controlled clinical trials on whether simvastatin can help prevent recurrent acute pancreatitis(RAP) and acute attack of CP, and avoid the natural progression of AP to CP is under way (121, 122). We expect that they can obtain gratifying results. Other experts have explored the relationship between statins and the incidence and prognosis of pancreatic cancer(PC): statins can reduce the risk of PC in high-risk groups and improve the survival rate of patients with grade I or II PC and locally resectable PC (123). In contrast, another Danish cohort study showed that statins was not associated with the risk of PC in patients with CP (120). The reason for the difference is due to different races or other influencing factors need to be further discussed.

Statins can slow down the progress of CP mainly because it can improve lipid metabolism and effectively fight inflammation. As a classic drug for the treatment of coronary atherosclerosis, it has been widely used in clinical. If its efficacy for CP can be determined, clinical application will be easier than other new drugs.

#### 3.4.7 Vitamin D

Vitamin D(VD) is an important steroid hormone regulating calcium and phosphorus levels in the body, and plays an important role in the treatment of bone diseases. Recent studies have also confirmed that it has multiple effects such as immune regulation, anti-inflammation and anti-fibrosis. VD deficiency is a common problem in patients with CP (124), but whether VD deficiency can be recognized as an independent risk factor of CP remains controversial.

*In vivo* and *in vitro* experiments have confirmed that VD and its analogues can inhibit the development of pancreatitis and pancreatic ductal adenocarcinoma(PDAC) by inhibiting the activation of PSCs (125, 126). Clinical trials show that oral or intravenous VD can improve VD deficiency in patients with CP, alleviate its related symptoms, and improve the quality of life of patients, but the effect is only shown when VD is deficient (127). When the deficiency is corrected, VD supplementation will no longer have a significant effect on patients.

VD can inhibit the development of CP mainly due to its anti-inflammatory, anti-fibrosis and immunomodulatory effects (128). In addition, VD can also inhibit the cell cycle of a variety of cells, inhibit cell proliferation, interfere with the dedifferentiation of tumor cells, and induce apoptosis (129–131), which is of great significance to prevent CP from developing into PC and the deterioration of PC (132).

## 4 Conclusions and future perspectives

With continuing research the risk factors and pathophysiological mechanisms of CP have been clarified, patient management guidelines have been standardized, and endoscopic technology and surgery have become important methods for treating CP complications in the late stages of the disease. It is unfortunate that current clinical treatments for CP are based on symptomatic treatment. At present, there are no effective means to prevent the progression of CP and improve pancreatic function. In addition, as a high-risk factor for pancreatic cancer, if the development of pancreatic fibrosis in patients with CP is not effectively controlled, they will be at a higher risk of carcinogenesis.

Here we have summarized the current progress in etiological treatment of CP from the aspects of regenerative medicine, traditional Chinese medicine, biological therapy, and immune- targeted therapy. Different drugs and treatment methods affect disease progression through some similar pathways, such as reducing the level of inflammatory factors and oxidative stress to improve the inflammatory state of the pancreas and prevent the occurrence of precancerous lesions. In addition, PSCs play an important regulatory role, and therefore, are excellent therapeutic targets for preventing the progression of pancreatic fibrosis.

Although it has been effectively validated in cell- and animal-based trials, most drugs and therapeutic methods have not yet entered clinical trials. In view of the increasing disease burden of CP and PC, we should encourage further *in vivo* and *in vitro* experiments to provide more reliable evidence for the early application of effective treatment methods in clinical practice.

## Author contributions

B-QL and X-YLiu reviewed literature and originally drafted the manuscript. TM, T-HZ, PZ, QZ, and YZ contributed to editing and embellished the manuscript. X-YLi approved the final version of the manuscript. All authors contributed to the article and approved the submitted version.

## Funding

National Natural Science Foundation of China (Grant 82270676), 2021Shandong Province Graduate Education and Teaching Reform Research Project (SDYJG21110), Qingdao Chinese Medicine Technology Project (2021-zyym26).

## Acknowledgments

We would like to thank Editage (www.editage.com) for English language editing. We thank all the authors for helping with the writing and publication of this article.

## Conflict of interest

The authors declare that the research was conducted in the absence of any commercial or financial relationships that could be construed as a potential conflict of interest.

## Publisher’s note

All claims expressed in this article are solely those of the authors and do not necessarily represent those of their affiliated organizations, or those of the publisher, the editors and the reviewers. Any product that may be evaluated in this article, or claim that may be made by its manufacturer, is not guaranteed or endorsed by the publisher.
